# Association between *TP53* mutation and high 21-gene recurrence score in estrogen receptor-positive/HER2-negative breast cancer

**DOI:** 10.1038/s41523-022-00384-3

**Published:** 2022-02-16

**Authors:** Jung Hwan Ji, Soong June Bae, Kyungsoo Kim, Chihhao Chu, Kyung-A Lee, Yoonjung Kim, Jee Hung Kim, Joon Jeong, Sung Gwe Ahn

**Affiliations:** 1 Department of Surgery, Gangnam Severance Hospital, Yonsei University College of Medicine, Seoul, Republic of Korea; 2 Institute for Breast Cancer Precision Medicine, Yonsei University College of Medicine, Seoul, Republic of Korea; 3 Department of Laboratory Medicine, Gangnam Severance Hospital, Yonsei University College of Medicine, Seoul, Republic of Korea; 4 Division of Medical Oncology, Department of Internal Medicine, Gangnam Severance Hospital, Yonsei University College of Medicine, Seoul, Republic of Korea

**Keywords:** Breast cancer, Tumour biomarkers

## Abstract

We investigated the association between *TP53* mutation and 21-gene recurrence score (RS) in ER-positive/HER2-negative breast cancer (BC) using data from 141 patients who underwent *TP53* sequencing and Oncotype DX^®^ tests. We detected *TP53* mutations in 18 (12.8%) patients. Most patients with *TP53* mutation had a high 21-gene RS (≥26). The average 21-gene RS was higher in *TP53* mutant tumors. Multivariate analysis showed that mutated *TP53* is an independent factor for a high 21-gene RS. Mutated *TP53* remained closely associated with high 21-gene RS in patients with low pathological risk (*n* = 103). In the ER^+^/PR^+^/HER2-negative subset (*n* = 356) of The Cancer Genome Atlas, the non-luminal A intrinsic subtype was more prevalent in the group with mutant *TP53*. mRNA levels of p53-regulated senescence gatekeeper and cell cycle-related genes were increased in BC with mutated *TP53*. Mutational analysis of *TP53* helped identify endocrine-resistant tumors.

## Introduction

The tumor suppressor protein p53 can induce cell cycle arrest, apoptosis, senescence, or ferroptosis in response to stress signals^1,2^, and it prevents the accumulation of cancer-causing mutations that lead to the development of malignant tumors^3^. Approximately 30% of the breast tumors harbor a *TP53* mutation^4^, and the frequency, spectrum, and timing of these mutations vary with the molecular subtype of the disease^5^. Although the prevalence of *TP53* mutations is lower in luminal than in basal-like tumors^6^, *TP53* mutation is the second most common mutation in the luminal type^7^.

*TP53* mutation is a poor prognostic factor in hormone receptor (HR)-positive luminal tumors^8–10^. Moreover, 11 (38%) of the 29 aromatase inhibitor-resistant, estrogen receptor (ER)-positive (^+^) tumors had somatic mutations in genes involved in the *TP53* pathway, including *TP53, ATR, APAF1*, or *THBS1*^7^, indicating an association between *TP53* mutations and endocrine resistance. Somatic *TP53* mutations are more prevalent in patients with primary than in patients with secondary endocrine-resistant or -responsive ER^+^ metastatic breast cancer (BC)^11^.

The Oncotype DX^®^ 21-gene recurrence score (RS) is a validated multigene signature for predicting outcomes and guiding chemotherapy in ER^+^/HER2^−^ BC^12–14^. This score has also been evaluated in cohorts given neoadjuvant endocrine therapy or chemotherapy. Patients with a high 21-gene RS respond poorly to neoadjuvant endocrine therapy^15–17^, implying an association between RS and endocrine resistance. The pathological complete response rates in neoadjuvant chemotherapy cohorts are higher when tumors have a high 21-gene RS^18,19^. Our translational study revealed that tumors with high 21-gene RS are more chemo-sensitive, based on chemoresponse assays in vitro^20^. These clinical and translation studies suggest an association between high 21-gene RS and endocrine resistance in ER^+^ BC.

A correlation between the *TP53* mutation and 21-gene RS in ER^+^ BC has not yet been identified. We, therefore, investigated the association between the *TP53* mutation and the 21-gene RS in patients with ER^+^/HER2^−^ BC and evaluated the molecular characteristics of ER^+^/HER2^−^ BC with the *TP53* mutation using The Cancer Genome Atlas (TCGA) database.

## Results

### Characteristics of patients with *TP53* mutations

**Fig. 1:**
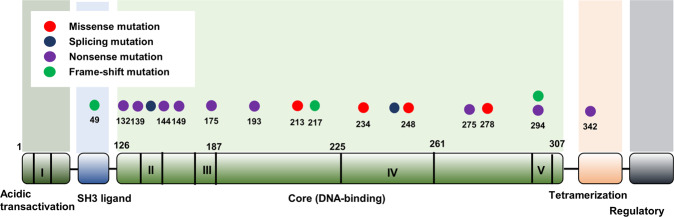
Types and locations of the *TP53* mutations in the 18 patients from the mutant *TP53* group. Nonsense mutations of *TP53* were most prevalent and were frequently located in exon 5. Each circle indicates patients with *TP53* mutation (*n* = 18) and the color of circle indicates the type of mutation. The number beneath each circle indicates the location of *TP53* mutation.

We identified *TP53* gene mutations in 18 (12.8%) of 141 patients with 21-gene RS and a sequenced *TP53* gene and studied their types and locations. Nonsense and missense *TP53* mutations were the most prevalent types in 9 (50%) and 4 (22.2%) of 18 patients, respectively (Fig. 1). The most frequent locations of these mutations were exons 5 and 8 in 5, (27.8%), and (4 (22.2%) of the 18 patients (Fig. 1). We also detected mutations in introns of the *TP53* gene in 2 (11.1%) patients.

We investigated the clinicopathological features according to *TP53* mutational status (Table 1). The median age was higher in the group with mutated, than wild-type *TP53* (57.5 vs. 47.0 y, *p* = 0.091; Table 1). Mutated *TP53* was significantly associated with menopausal status (*p* = 0.005), T stage (*p* = 0.021), histological grade (HG) (*p* = 0.002), lymphovascular invasion (LVI) (*p* = 0.018), and Ki-67 levels (*p* ≤ 0.001). Mutations of *TP53* were more frequent among postmenopausal patients with a higher histological grade, Ki-67 > 20%, larger tumors, and LVI. Chemotherapy was more frequently administered to patients with, than without the *TP53* mutation (*p* < 0.001; Table 1).

**Table 1:** Baseline patient characteristics and treatments.

Variables	TP53 mutation− (*n* = 123)	TP53 mutation+ (*n* = 18)	Total (*n* = 141)	*P*^a^
Median age (*y*; range)	47 (26–75)	57.5 (41–63)	48 (41–75)	0.091^b^
Menopausal status				0.005^c^
Premenopausal	77 (62.6%)	5 (27.8%)	82 (58.2%)	
Postmenopausal	46 (37.4%)	13 (72.2%)	59 (41.8%)	
Estrogen receptor^d^				0.652
Low	10 (8.1%)	2 (11.1%)	12 (8.5%)	
High	113 (91.9%)	16 (88.9%)	129 (91.5%)	
Progesterone receptor				0.475
Negative	17 (13.8%)	4 (22.2%)	21 (14.9%)	
Positive	106 (86.2%)	14 (77.8%)	120 (85.1%)	
T stage				0.021^c^
1	70 (56.9%)	5 (27.8%)	75 (53.2%)	
2 or 3	53 (43.1%)	13 (72.2%)	66 (46.8%)	
N stage				0.773
0	93 (75.6%)	13 (72.2%)	106 (75.2%)	
1 or 2	30 (24.4%)	5 (27.8%)	35 (24.8%)	
Histologic grade				0.002
1 or 2	111 (91.0%)	11 (61.1%)	122 (87.1%)	
3	11 (9.0%)	7 (38.9%)	18 (12.9%)	
Nuclear grade				0.329
1 or 2	101 (82.8%)	13 (72.2%)	114 (81.4%)	
3	21 (17.2%)	5 (27.8%)	26 (18.6%)	
LVI^e^				0.018
Negative	96 (78.0%)	9 (50.0%)	105 (74.5%)	
Positive	27 (22%)	9 (50.0%)	36 (25.5%)	
Ki-67				<0.001
≤ 20	119 (96.7%)	12 (66.7%)	131 (92.9%)	
< 20	4 (3.3%)	6 (33.3%)	10 (7.1%)	
Chemotherapy				<0.001
No	99 (80.5%)	5 (27.8%)	104 (73.8%)	
Yes	24 (19.5%)	13 (72.2%)	37 (26.2%)	
Endocrine therapy				0.117
Tamoxifen	72 (58.5%)	6 (33.3%)	78 (55.3%)	
AI	50 (40.7%)	12 (66.7%)	62 (44.0%)	
Fulvestrant	1 (0.8%)	0 (0.0%)	1 (0.7%)	
Radiotherapy				0.296^c^
No	50 (40.7%)	5 (27.8%)	55 (39.0%)	
Yes	73 (59.3%)	13 (72.2%)	86 (61.0%)	

^a^Fisher exact test.

^b^Mann–Whitney U test.

^C^χ2 test.

^d^High, Allred score 5–8; Low, Allred score 2–4.

^e^Lymphovascular invasion.

### Mutant *TP53* and Oncotype Dx^®^ 21-gene RS

**Fig. 2:**
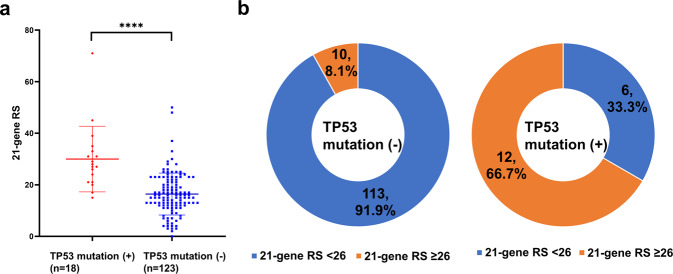
Association between *TP53* mutation and Oncotype DX® recurrence scores (RSs). **a** Mean Oncotype DX^®^ RS according to mutated *TP53* compared using Student’s *t*-tests (*p* < 0.001). Error bars correspond to standard error of the mean. **b** Distribution of Oncotype DX^®^ risk group compared based on *TP53* mutation using Fisher’s exact tests (*p* = 0.021). Oncotype DX^®^ RS ≥ 26 indicates high risk.

The average 21-gene RSs were 30 in and 16.41 in groups with mutant and wild-type *TP53*, respectively (Fig. 2a). The 21-gene RS was more likely to be ≥ 26 in tumors with—than without—a *TP53* mutation (*p* = 0.021; Fig. 2b).

**Fig. 3:**
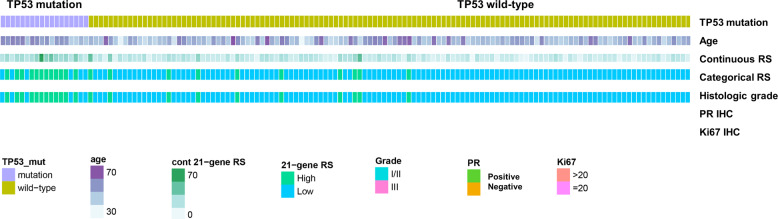
Heatmap with 21-gene recurrence score and sequenced *TP53* gene (*n* = 141). Median age and 21-gene recurrence scores based on *TP53* mutation compared using Mann–Whitney U tests. Distribution of Oncotype DX risk group compared according to mutated *TP53* using χ^2^ tests. Histological grade, progesterone receptor status, and Ki-67 expression were compared using Fisher’s exact tests.

We identified factors associated with a high 21-gene RS using binary logistic regression analysis. Univariate analyses showed that T stage, progesterone receptor (PR), HG, Ki-67 levels, and *TP53* mutation were significant (Table 2), and multivariate analysis revealed *TP53* mutation, T stage, and PR as independent variables that were associated with a high 21-gene RS (Table 2). The odds ratio (OR) of the *TP53* mutation was 21.632 (95% confidence interval [CI], 5.877–79.627). Figure 3 shows the differences between the 21-gene RS and other pathological variables based on the *TP53* mutation.

**Table 2:** Binary logistic regression analysis of factors associated with high (≥26) Oncotype DX^®^ recurrence score.

Variables	Univariate		Multivariate	
	Odds ratio (95% CI)	*P*	Odds ratio (95% CI)	*P*
T stage				
1	Ref		Ref	
2 or 3	3.680 (1.345–10.067)	0.011	4.262 (1.161–15.646)	0.029
N stage				
Negative	Ref			
Positive	0.261 (0.058–1.177)	0.08		
Estrogen receptor^a^				
High	Ref			
Low	1.090 (0.222–5.352)	0.915		
Progesterone receptor				
Positive	Ref		Ref	
Negative	4.659 (1.643–13.212)	0.004	9.214 (2.275–37.311)	0.002
Histologic grade				
1 or 2	Ref		-	-
3	6.171 (2.088–18.239)	0.001	-	-
Nuclear grade				
1 or 2	Ref			
3	2.432 (0.874–6.761)	0.089		
Ki-67				
≤20	Ref		-	-
>20	10.781 (2.742–42.385)	0.001	-	-
LVI^b^				
Negative	Ref			
Positive	1.112 (0.399–3.102)	0.839		
TP53 mutation				
Negative	Ref		Ref	
Positive	22.600 (6.986–73.116)	<0.001	21.632 (5.877–79.627)	<0.001

^a^High, Allred score 5–8; Low, Allred score 2–4.

^b^Lymphovascular invasion.

### *TP53* mutation in subset with good pathologic features

We investigated the relationship between mutated *TP53* and the 21-gene RS in the subset with good pathological features. The 21-gene RS can determine patients who are eligible for chemotherapy among those with favorable pathological parameters who are nevertheless at high risk of relapse. We compared the clinical characteristics of 103 patients who were PR-positive and had low HG (I/II) and low (≤20%) Ki-67 expression based on *TP53* mutations (Supplementary Table 1). Median age, menopausal status, and T stage were altered by the *TP53* mutation, whereas other pathological parameters were not.

Tumors in this subset with the *TP53*-mutant had a higher mean 21-gene RS and an elevated high 21-gene RS rate (Supplementary Fig. 1). Our multivariate analysis selected the *TP53* mutation as the sole significant factor for a high 21-gene RS (Supplementary Table 2).

### Distant recurrence-free survival according to mutated *TP53*

**Fig. 4:**
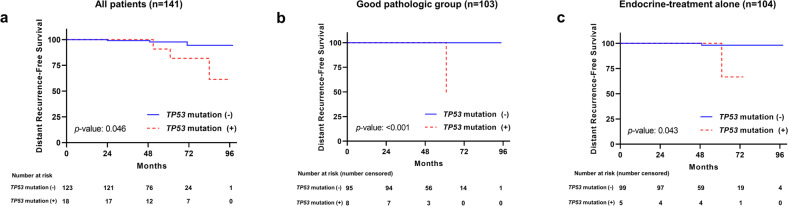
Kaplan–Meier survival plots for distant recurrence-free survival (DRFS) based on *TP53* mutation. Rates of DRFS according to *TP53* mutations in **a** all patients, in **b** subset with good pathological (PR^+^/low HG/low Ki-67) outcomes, and in **c** patients given endocrine treatment alone (*p* = 0.046, *p* < 0.001 and *p* = 0.043, respectively; log-rank tests).

We analyzed distant recurrence-free survival (DRFS) to determine the prognostic value of mutated *TP53*. Six patients had distant recurrence at a median follow-up of 51 (6–98) months. Figure 4a shows that the DRFS was significantly lower in the group with, than without a *TP53* mutation (*p* = 0.046). In addition, the results were consistent; the DRFS significantly differed according to the *TP53* mutation in a subset with good pathological features (Fig. 4b; *n* = 103, *p* < 0.001) and in a group that had received only endocrine therapy (Fig. 4c; *n* = 104, *p* = 0.046). However, the DRFS did not significantly differ according to 21-gene RS ≥ 26 vs. < 26 (Supplementary Fig. 2).

### Mutant *TP53* in HR^+^/HER2^−^ stage I/II BC tumors from TCGA

Because mutant *TP53* was associated with a high 21-gene RS in the subset with good pathological features, we investigated the molecular characteristics of mutated *TP53* in ER^+^/PR^+^/HER2^−^, stage I/II BC tumors using TCGA database.

**Fig. 5:**
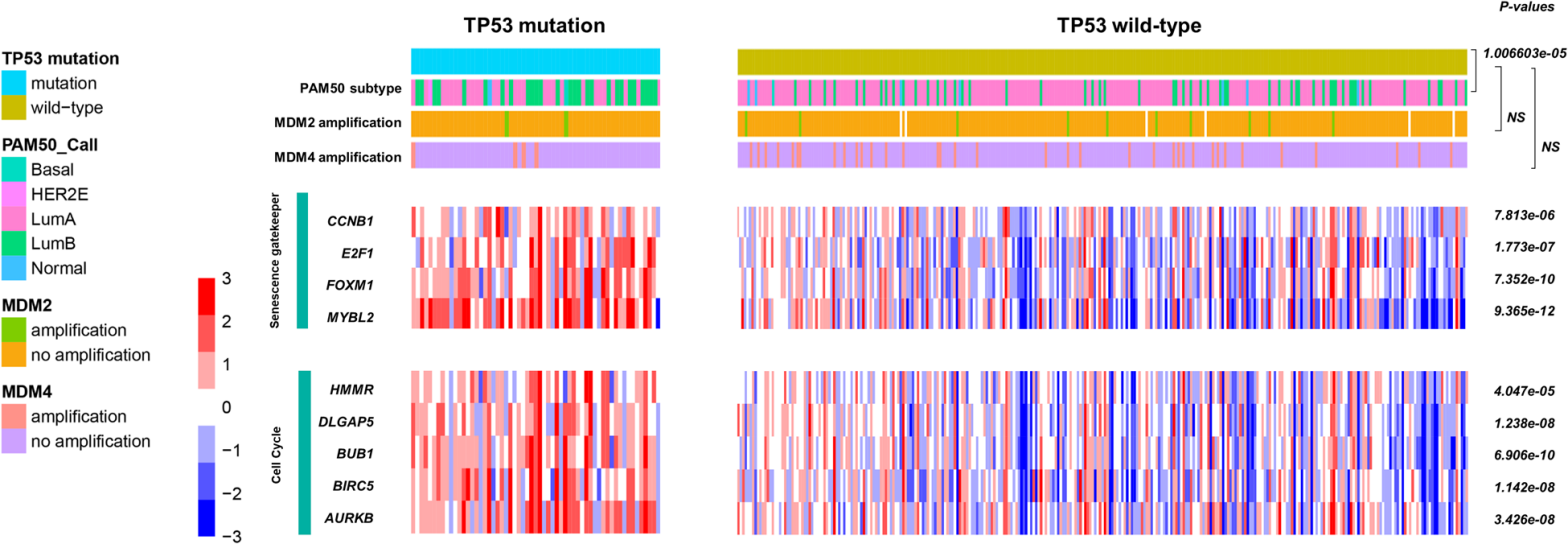
PAM50 subtypes, MDM2/4 amplification, and *TP53*-associated gene expression based on *TP53* mutation in 356 ER^+^/PR^+^/HER2^−^ samples in TCGA (*n* = 365). PAM50 subtype distribution, MDM2/4 amplification, and mRNA expression according with *TP53* mutation were compared. The *p*-values were obtained using χ2 tests, Fisher’s exact tests, and Student’s t-tests, respectively.

We identified 356 patients with ER^+^/PR^+^/HER2^−^, stage I/II BC tumors in TCGA database (Fig. 5 and Supplementary Table 3). We compared those with defined PAM50 subtypes. The prevalence of the luminal A subtype was higher in the group with wild-type *TP53*, whereas that of luminal B, HER2-enriched, and basal-like tumors, was higher in the group with mutant *TP53* (*p* < 0.001). We then compared *MDM2/4* amplification between the two groups, because *MDM2/4* is a p53-specific E3 ubiquitin ligase that limits the p53 growth-suppressive function in unstressed cells^21,22^. The frequency of *MDM2/4* amplification was comparable between the groups.

We compared the mRNA levels of p53-regulated, senescence gatekeeper, and cell cycle genes. The senescence gatekeeper genes, *CCNB1, E2F1, FOXM1*, and *MYBL2*, and the cell cycle-associated genes, *HMMR, DLGAP5, BUB1, BIRC5*, and *AURKB*, were upregulated in the group with mutant *TP53* (Fig. 5).

## Discussion

To the best of our knowledge, this is the first study to investigate the relationship between mutant *TP53* and the 21-gene RS. We associated mutant *TP53* with a high 21-gene RS (≥ 26) in ER^+^/HER2^−^ BC. The average 21-gene RS and the frequency of high categorical 21-gene RS were significantly higher in the group with mutant, than wild-type *TP53*. Therefore, having a *TP53* mutation was an independent factor for a high 21-gene RS.

The association between the two markers was reproducible in pathologically low-risk subgroups such as PR^+^/histological grade (HG) 1/2, and low Ki-67 subgroups. The 21-gene RS helps to identify patients with ER^+^ BC who are at genomic high and low risk^13,23^. Mutant *TP53* or a high 21-gene RS can both function as biomarkers for endocrine resistance^7,11,15–17,24–26^. Our findings showed that *TP53* mutational analysis could help to detect endocrine-resistant tumors in patients with good pathological features.

We compared the molecular characteristics of early ER^+^/PR^+^/HER2^-^ BC tumors based on *TP53* mutational status using TCGA database. Because the 21-gene RS has been principally evaluated in early ER^+^ BC with limited nodal involvement^13,27^, we included stage I/II BC. Among the defined PAM50 subtypes, we found that *TP53* mutant tumors had non-luminal A (low endocrine sensitivity, chemosensitive) rather than luminal A (endocrine-sensitive) subtypes^28–31^. This indicated that *TP53* mutant tumors are likely to be endocrine resistant.

We also quantified the mRNA levels of p53-regulated genes associated with the cell cycle or transcription. Upregulated *TP53* induces or represses many genes involved in cell cycle regulation, DNA repair, apoptosis, and senescence^2,32^. Our finding of upregulated gene clusters in the mutant *TP53* group indicated that *TP53*-mutant, ER^+^ tumors are highly proliferative and aggressive. The upregulated *CCNB1* and *MYBL2* genes are among those listed for the 21-gene RS assay^33^. This, to some extent, supports an association between the two biomarkers. Further molecular studies are required to validate an association between genes in the 21-gene RS and those in the p53 signaling pathway.

The amplification rates of *MDM2/4* were comparable in tumors with wild-type and mutant *TP53*. One study found a slightly higher frequency of *MDM2* amplification in tumors with mutant, than wild-type *TP53*^34^. However, that study considered BC regardless of subtypes, whereas we investigated only ER^+^/PR^+^/HER2^−^ BC. Considering ongoing efforts to target *MDM2/X* in breast cancer^35,36^, an association between *MDM2/4* amplification and *TP53* mutational status should be further explored.

A major limitation of the present study was the small sample size in the institutional database and the absence of information on 21-gene RS in TCGA. Due to the small patient cohort and a short follow-up, a prognostic assessment using mutant *TP53* combined with 21-gene RS was not obtainable. Whether adding information about mutant *TP53* to the 21-gene RS could improve the prognostic ability should be evaluated in a larger patient cohort. Biological and clinical characteristics were not sorted based on the location and type of *TP53* mutation^37^. Nevertheless, our findings underscored the fact that *TP53* is associated with a high 21-gene RS in ER^+^/HER2^−^ BC, and *TP53* mutational analysis detected endocrine-resistant tumors.

We concluded that the clinical and molecular evidence points toward an association between mutated *TP53* and a high 21-gene RS in terms of endocrine resistance. By detecting *TP53* mutation, endocrine-resistant tumors could be identified even in patients who had good pathological features.

## Methods

### Patients

This study was approved by the Institutional Review Board of Gangnam Severance Hospital (3-2021-0296) and adhered to the clinical practice guidelines of the Declaration of Helsinki (2013 amendment). In all patients, informed consent form for research of human-derived material was obtained. We retrospectively evaluated 572 patients who were surgically treated for ER^+^/HER2^−^ primary invasive BC who had undergone Oncotype DX^®^ tests at Gangnam Severance Hospital, Yonsei University College of Medicine, Seoul, Korea, between August 2011 and March 2020. Among the 572 patients, we included 141 whose *TP53* gene had been sequenced. Clinicopathological data were extracted from electronic medical records and included age, menopausal status, ER and PR status, tumor size, nodal status, HG, nuclear grade (NG), LVI, Ki-67, Oncotype DX^®^ 21-gene RS, *TP53* mutation status, and some *TP53* mutation characteristics. We excluded patients diagnosed with recurrent or metachronous BC. Tumors were staged according to the 7th edition of the American Joint Committee on Cancer. Tissue sections were histologically assessed using the Elston-Ellis modification of the Scarff−Bloom−Richardson grade. Adjuvant systemic therapy and/or radiotherapy were administered according to standard guidelines based on the age of the patients, tumor characteristics, and axillary lymph node status. Endocrine therapy was administered to all patients.

We used specific ER antibodies (1:100 clone 6F11; Novocastra, Newcastle upon Tyne, UK) and the progesterone receptor (PR; clone 16; Novocastra) for immunohistochemistry (IHC)^38^. The IHC results for ER and PR were stratified based on modified Allred scoring, in which scores of 0–1, 2–4, 5–6, and 7–8 represented negative, weak, moderate, and strong expression, respectively^39^. Groups with strong and moderate ER expression were considered ER-high and groups with weak expression were considered ER-low. Scores from 2 to 8 (weak to strong) were all considered PR positive.

### *TP53* gene sequencing

We determined genomic variants using a next-generation sequencing (NGS) panel of 143 genes, including *TP53*. Among 141 patients, we analyzed 69 (48.9%) using polymerase chain reaction (PCR)-denaturing high-performance liquid chromatography (DHPLC), and 72 (51.1%) using targeted NGS (Supplementary Fig. 3). We analyzed *TP53* mutations using PCR-DHPLC and direct sequencing until December 2016 and targeted NGS thereafter.

Mutations in exons 5–9 of the *TP53* gene were analyzed by PCR as described^40,41^, using primers designed to amplify the exons and flanking introns of the *TP53* gene^40^. Amplification proceeded using Accu-Power™ Premix (Bioneer, Daejeon, Korea) under the following cycling conditions: 94 °C for 4 min, 50 cycles of 94 °C for 1 min, 60 °C for 30 s, and 72 °C for 30 s, and then 72 °C for 15 min. Purified PCR products obtained using QIAquick^®^ Gel Extraction kits (Qiagen, Düsseldorf, Germany) were sequenced using BigDye™ Terminator Cycle Sequencing Ready Reaction kits (Applied Biosystems, Foster City, CA, USA) under the following cycling conditions: 96 °C for 5 min, 24 cycles at 96 °C for 10 s, 50 °C for 5 s, and 60 °C for 4 min, then 72 °C for 5 min. The sequences were analyzed using an ABI 3500Dx system (Thermo Fisher Scientific Inc.). Forward and reverse strands were sequenced to confirm mutations.

We applied targeted NGS using an Oncomine™ comprehensive panel and an Ion Torrent™ S5 XL system (Thermo Fisher Scientific Inc.). We extracted DNA from fresh tissues and determined its yield and quality using Torrent v. 5.2 and Ion Reporter™ v. 5.2 (both from Thermo Fisher Scientific Inc.). Genomic variants were analyzed using an NGS panel of 143 genes, including *TP53*. Supplementary Figure 4 shows other genomic alterations in 72 patients assessed using targeted NGS.

### Oncotype Dx^®^ assays

We calculated 21-gene RSs using Oncotype Dx^®^ assays^23,33^. RSs are derived from the reference-normalized expression of 16 genes associated with cancer (*Ki-67, STK15, Survivin*, [*BIRC5*]*, CCNB1* [*cyclin B1*]*, MYBL2, GRB7, HER2, ER, PGR, BCL2, SCUBE2, MMP11* [*stromelysin 3*]*, CTSL2* [*cathepsin L2*]*, GSTM1, CD68*, and *BAG1*) and five reference genes, β-actin (*ACTB*), *GAPDH, GUS, RPLPO*, and *TFRC*, and then calculated on a scale of 0–100. Quantitative single gene scores were determined by reverse transcriptase-PCR. The expression of each gene was measured in triplicate and normalized to the reference genes. Oncotype Dx^®^ assays of RNA extracted from formalin-fixed paraffin-embedded tissues proceeded at Genomic Health Inc. (Redwood City, CA, USA). We defined scores >26 as high 21-gene RSs according to the TAILORX trial^13^.

### TCGA data

We identified HR^+^/HER2^−^ tumors in TCGA database using immunohistochemistry (IHC) for ER, PR, and HER2 and RNA-seq data as described^42,43^ and normalized, log2-transformed, and median-centered expression. Information about *TP53* mutations and genomic variants was downloaded from the cBioPortal^44^. We identified PAM50-defined intrinsic subtypes as luminal A, luminal B, HER2-enriched, basal-like, and normal-like^43,45^, and compared the subtype distribution and *MDM2/4* amplification between the groups with mutant and wild-type *TP53*. We also quantified the expression of p53-regulated senescence gatekeeper and cell cycle genes^35^.

### Statistical analysis

Continuous variables were compared using Mann–Whitney U tests and Student’s *t*-tests. The normal distribution of continuous variables was assessed using Kolmogorov–Smirnov tests. Nominal variables were compared using χ^2^ or Fisher exact tests. Predictive factors for high 21-gene RS (≥26)^13^ were identified by multivariate binary logistic regression analysis of all variables. Variables with *p* < 0.05 were included in the multivariate model, and the final model was realized using backward stepwise (Wald) selection. We analyzed survival using DRFS defined as the interval from curative surgery to the first distant recurrence or last censored. Survival was determined using Kaplan–Meier plots, and two groups were compared using log-rank tests. All data were statistically analyzed using SPSS version 25.0 (IBM Corp, Armonk, NY, USA) and R software (https://www.r-projet.org; version 3.6.1). Values with *p* < 0.05 were considered statistically significant.

### Reporting summary

Further information on research design is available in the Nature Research Reporting Summary linked to this article.

## Supplementary information

Supplementary information

Reporting Summary

## Data Availability

The TCGA data analyzed within this study are described in the following data record: 10.1038/nature11412 (2012), 10.1016/j.cell.2015.09.033 (2015) and 10.1126/scisignal.2004088 (2013)^42–44^. The somatic mutations and gene amplification data are included as Supplementary Table 4 and presented in Supplementary Fig 4. For more data access requests, please contact the corresponding author, Dr. Sung Gwe Ahn.
