# Supplementary material for: Association between TP53 mutation and high 21-gene recurrence score in estrogen receptor-positive/HER2-negative breast cancer
Source: NPJ Breast Cancer. 2022 Feb 16;8:19. doi: 10.1038/s41523-022-00384-3 (PMC8850427; doi:10.1038/s41523-022-00384-3)
Supplement: Supplementary file 1 — Supplementary information [file 41523_2022_384_MOESM1_ESM.pdf]

Supplementary Fig. 1 Association between *TP53* mutation and Oncotype DX® recurrence score (RS) (PR+/low-HG/low-Ki-67). a Means of Oncotype DX® RS according to the *TP53* mutation were compared using the Student's *t*-test (****p*-value <0.001). Error bars correspond to standard error of the mean. **b** Oncotype DX® risk group distribution according to *TP53* mutation. The distributions of the Oncotype DX® risk group were compared based on the *TP53* mutation using the Fisher's exact test ($p<0.001$).

a

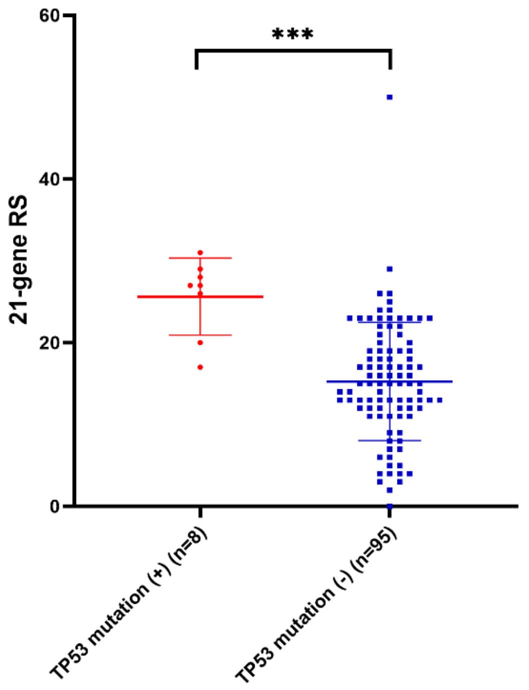

b

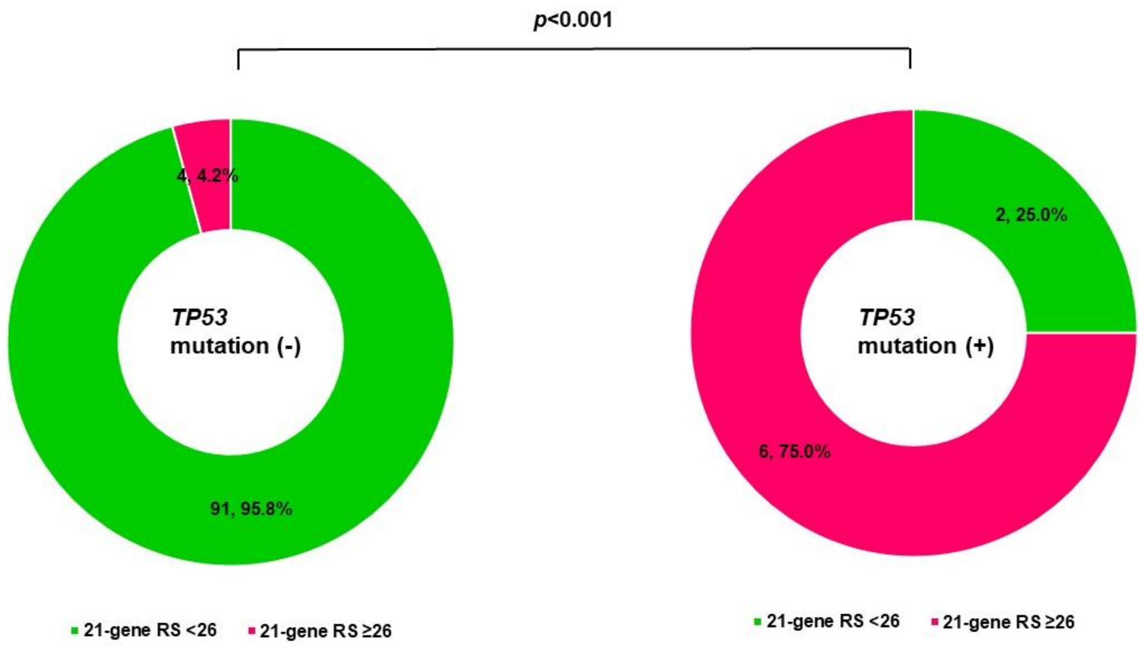

Supplementary Fig. 2 Kaplan-Meier survival plots of distant recurrence-free survival (DRFS) based on binary 21-gene recurrence score (RS) (≥ 26 vs. < 26). Distant recurrence-free survival does not significantly differ according to 21-gene RS ($p = 0.11$).

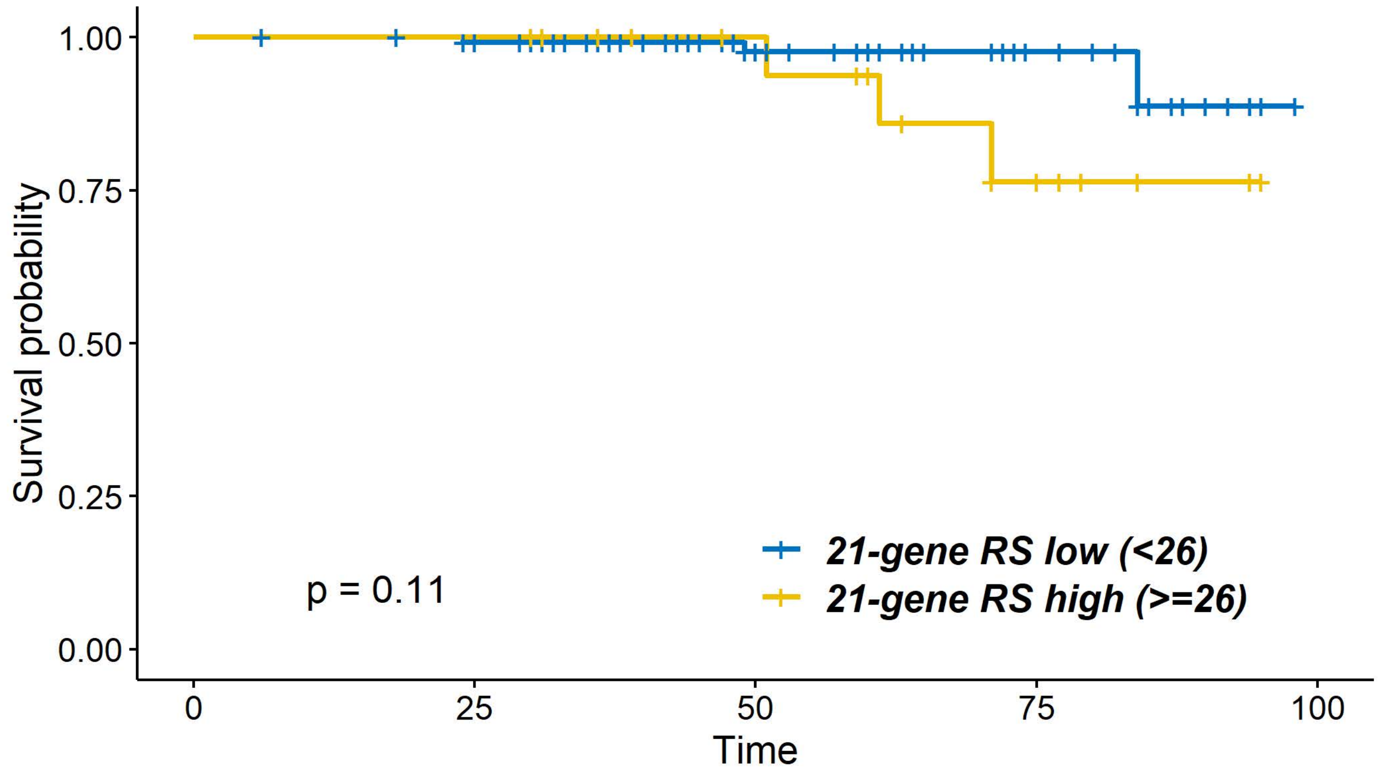

Number at risk (number censored)

	0	25	50	75	100
21-gene RS low (<26)	119 (0)	114 (6)	60 (62)	15 (102)	0 (116)
21-gene RS high (>=26)	22 (0)	22 (0)	16 (6)	7 (13)	0 (19)

Supplementary Fig. 3 Consort diagram. Among 141 patients, 69 and 72 were respectively analyzed using polymerase chain reaction (PCR)-denaturing high-performance liquid chromatography (DHPLC), and targeted next-generation sequencing (NGS).

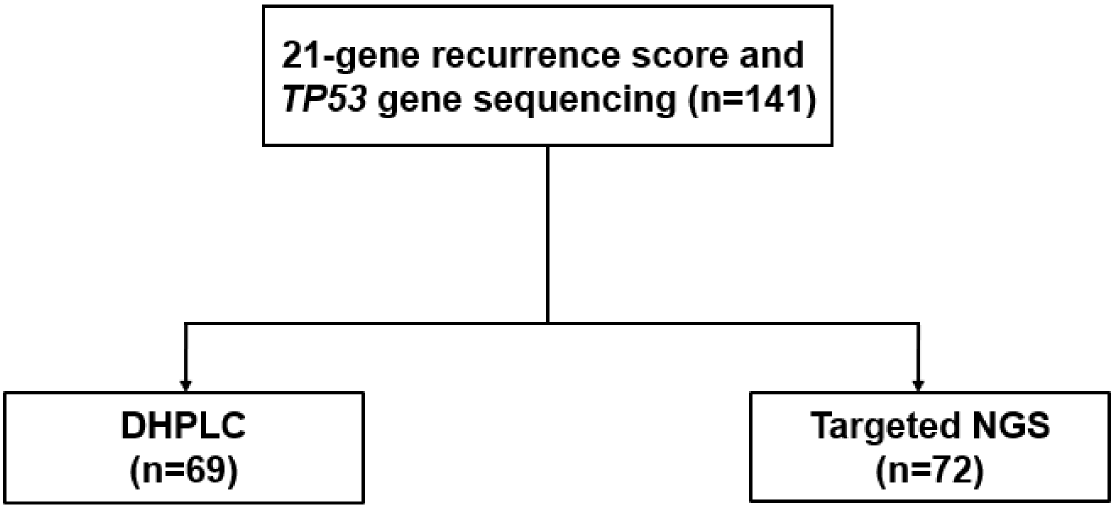

Supplementary Fig. 4 Genomic spectrum of 72 patients determined using targeted NGS. First row indicates status of *TP53* mutation. *PIK3CA* mutation is the most common, with 31 (43.1%) of the 72 patients in the cohort harboring this mutation. Among seven patients with *TP53* mutations, four (57.1%) also had a mutated *PIK3CA* gene. Each vertical line indicates one patient.

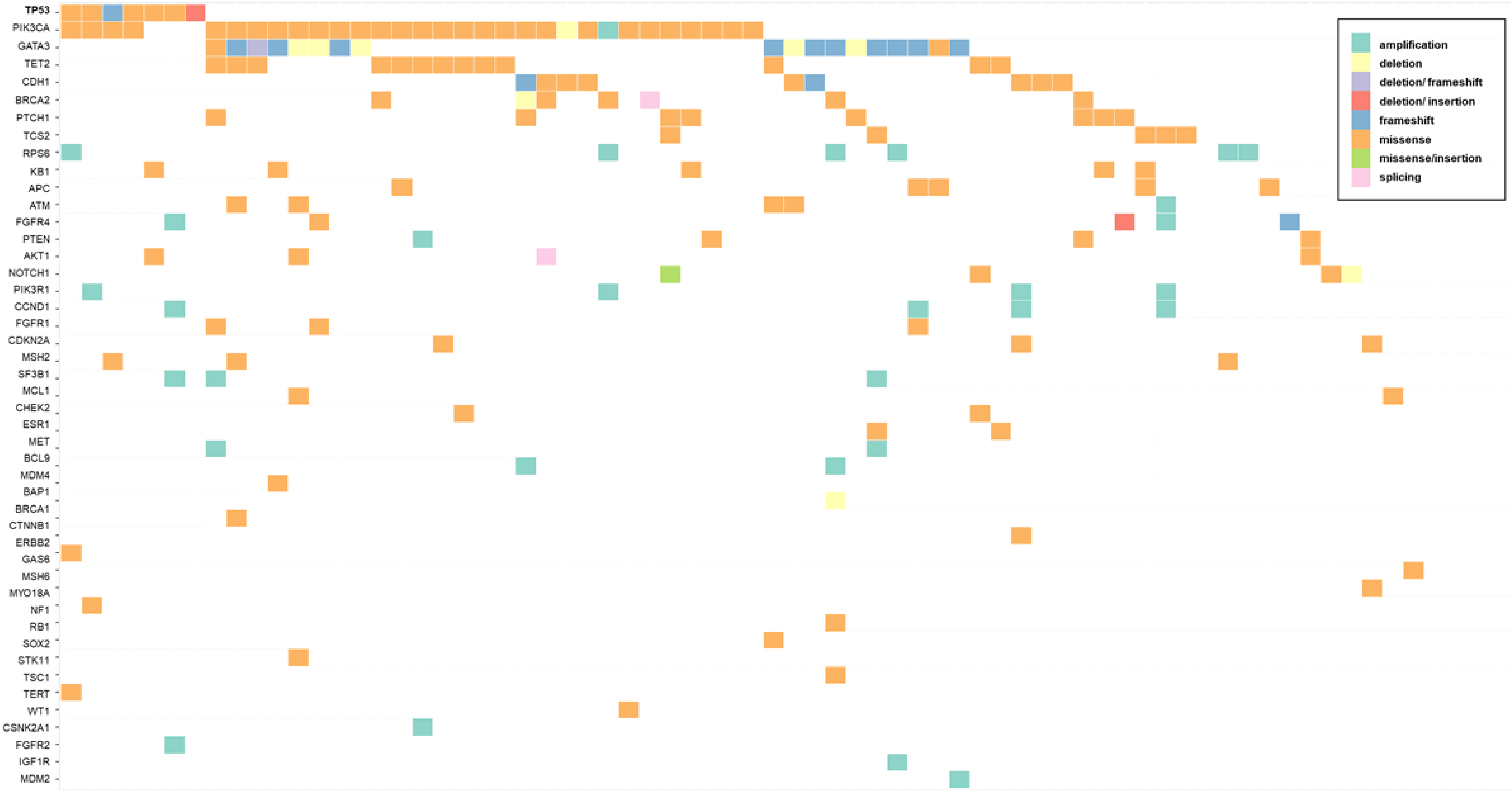

Supplementary Table 1. Baseline characteristics of patients (PR^+/low-HG/low-Ki-67).

Variables	TP53 mutation- (n=95)	TP53 mutation+ (n=8)	Total (n=103)	P[a]
Median age (y; range)	47.0 (26–75)	59.5 (45–63)	47.0 (26–75)	0.006[b]
Menopausal status				0.004
Premenopausal	64 (67.4%)	1 (12.5%)	65 (63.1%)	
Postmenopausal	31 (32.6%)	7 (87.5%)	38 (36.9%)	
Estrogen receptor[c]				0.532
Low	8 (8.4%)	1 (12.5%)	9 (8.7%)	
High	87 (91.6%)	7 (87.5%)	94 (91.3%)	
T stage				0.031
1	51 (53.7%)	1 (12.5%)	52 (50.5%)	
2 or 3	44 (46.3%)	7 (87.5%)	51 (49.5%)	
N stage				0.701
0	66 (69.5%)	5 (62.5%)	71 (68.9%)	
1 or 2	29 (30.5%)	3 (37.5%)	32 (31.1%)	
Nuclear grade				0.591
1 or 2	82 (86.3%)	8 (100.0%)	90 (87.4%)	
3	13 (13.7%)	0 (0.0%)	13 (12.6%)	
LVI[d]				0.202
Negative	72 (75.8%)	4 (50.0%)	76 (73.8%)	
Positive	23 (24.2%)	4 (50.0%)	27 (26.2%)	
Chemotherapy				<0.001
Not done	82 (86.3%)	2 (25.0%)	84 (81.6%)	
Done	13 (13.7%)	6 (75.0%)	19 (18.4%)	
Endocrine therapy				0.008
Tamoxifen	61 (64.2%)	1 (12.5%)	62 (60.2%)	
AI	33 (34.7%)	7 (87.5%)	40 (38.8%)	
Fulvestrant	1 (1.1%)	0 (0.0%)	1 (1.0%)	
Radiotherapy				0.141
Not done	40 (42.1%)	1 (12.5%)	41 (39.8%)	
Done	55 (57.9%)	7 (87.5%)	62 (60.2)	

[a]Fisher's exact test except [b]Mann–Whitney U test
[c]High, Allred score 5-8; Low, Allred score 2-4
[d]Lymphovascular invasion

Supplementary Table 2. Odds ratio (OR) and 95% confidential intervals (CIs) for high Oncotype DX® recurrence score (≥26) (PR+/low-HG/low-Ki-67).

Variables	Univariable OR (95% CI)	P	Multivariable OR (95% CI)	P
T stage				
1	Ref		-	-
2 or 3	4.651 (0.937–23.088)	0.060	-	-
N stage				
Negative	Ref			
Positive	0.525 (0.105–2.625)	0.433		
Estrogen receptor[a]				
High	Ref			
Low	N/A[b]			
Nuclear grade				
1 or 2	Ref			
3	0.860 (0.793–0.934)	0.355[b]		
LVI[c]				
Negative	Ref			
Positive	0.680 (0.135–3.422)	0.640		
TP53 mutation				
Negative	Ref		Ref	
Positive	68.250 (10.334–450.751)	<0.001	68.250 (10.334–450.751)	<0.001

[a]High, Allred score 5-8; Low, Allred score 2-4
[b]Not analysed due to no event
[c]Fisher's exact test
[d]Lymphovascular invasion

Supplementary Table 3. PAM50 subtype distribution and MDM2/4 amplification according to *TP53* mutation.

Variables	TP53 mutation- (n=297)	TP53 mutation+ (n=59)	Total (n=356)	P
PAM-50 subtype				<0.001[a]
Luminal A	234 (78.8%)	29 (49.2%)	263 (73.9%)	
Luminal B	55 (18.5%)	27 (45.8%)	82 (23.0%)	
HER2-enriched	0 (0.0%)	1 (1.7%)	1 (0.3%)	
Basal-like	1 (0.3%)	1 (1.7%)	2 (0.6%)	
Normal-like	7 (2.4%)	1 (1.7%)	8 (2.2%)	
MDM2[c]				0.999[b]
No amplification	281 (96.6%)	57 (96.6%)	338 (96.6%)	
Amplification	10 (3.4%)	2 (3.4%)	12 (3.4%)	
MDM4[c]				0.267[b]
No amplification	255 (87.6%)	55 (93.2%)	310 (88.6%)	
Amplification	36 (12.4%)	4 (6.8%)	40 (11.4%)	

[a]$\chi 2$ test
[b]Fisher's exact test
[c]Six missing values in the MDM2/4 amplification test

Supplementary Table 4. Somatic mutations and amplification identified in 141 patients

Patient ID	Cohort	Gene	cHGVS	pHGVS	Amplification
1	Target NGS	MSH6	c.1063G>A	p.Gly355Ser	No
2	Target NGS	PIK3CA	c.1663G>A	p.Glu545Lys	No
3	Target NGS	AKT	c.49G>A	p.Glu17Lys	No
4	Target NGS	PIK3R1	c.1770_1711delAAC	p.Lys567_Leu570del	No
4	Target NGS	CDH1	c.2494G>A	p.Val832Met	No
5	Target NGS	.	.	.	No
6	Target NGS	PIK3CA	c.3140A>G	p.His1047Arg	No
7	Target NGS	.	.	.	No
8	Target NGS	PIK3CA	c.1636C>A<br>c.3061T>C	p.Gln546Lys<br>p.Tyr1021His	No
9	Target NGS	.	.	.	No
10	Target NGS	PIK3CA	c.1625A>T<br>c.3204C>G	p.Glu542Val<br>p.Asn1068Lys	No
10	Target NGS	ESR1	c.1613A>G	p.Asp538Gly	No
11	Target NGS	PIK3CA	c.1633G>A	p.Glu545Lys	No
11	Target NGS	AKT1	c.49G>A	p.Glu17Lys	No
12	Target NGS	PIK3CA	c.3140A>G	p.His1047Arg	No
13	Target NGS	ERBB2	c.2332_2340dup	p.Gly778_Pro780dup	No
13	Target NGS	FGFR1	.	.	Yes
13	Target NGS	CCND1	.	.	Yes
14	Target NGS	PIK3CA	c.1634A>G	p.Glu545Gly	No
15	Target NGS	PIK3CA	c.353G>A	p.Gly118Asp	No
16	Target NGS	.	.	.	No
17	Target NGS	PIK3CA	c.3140A>G	p.His1047Arg	No
17	Target NGS	TP53	c.524G>A	p.Arg175His	No
17	Target NGS	CCND1	.	.	Yes
18	Target NGS	PIK3R1	c.1351_1356delGA	p.Glu451_Tyr452del	No
19	Target NGS	PIK3CA	c.328_330delGAA	p.Glu110del	No
20	Target NGS	PIK3CA	c.3140A>G	p.His1047Arg	No
20	Target NGS	PTEN	c.376G>A	p.Ala126Thr	No
21	Target NGS	PIK3R1	c.485G>T	p.Arg162Leu	No
22	Target NGS	.	.	.	No
23	Target NGS	PIK3CA	c.1035T>A	p.Asn345Lys	No
24	Target NGS	.	.	.	No
25	Target NGS	PIK3CA	c.1357G>A<br>c.353G>A<br>c.3140A>G	p.Glu453Lys<br>p.Gly118Asp<br>p.His1047Arg	No
26	Target NGS	PIK3CA	c.3140A>T	p.His1047Leu	No

27	Target NGS	PIK3CA	c.1633G>A	p.Glu545Lys	No
28	Target NGS	.	.	.	No
29	Target NGS	PIK3CA	c.1133G>A<br>c.3140A>G	p.Cys378Tyr<br>p.His1047Arg	No
30	Target NGS	PIK3CA	c.3140A>G	p.His1047Arg	No
30	Target NGS	TP53	c.396G>C	p.Lys132Asn	No
31	Target NGS	.	.	.	No
32	Target NGS	.	.	.	No
33	Target NGS	.	.	.	No
34	Target NGS	.	.	.	No
35	Target NGS	PIK3CA	c.3140A>G	p.His1047Arg	No
35	Target NGS	BRCA2	c.8488-1G>A	.	No
36	Target NGS	.	.	.	No
37	Target NGS	.	.	.	No
38	Target NGS	RB1	c.1578dup	p.Asp527Ter	No
38	Target NGS	BRCA1	17q21.31del	.	No
39	Target NGS	CHEK2	c.1111C>T	p.His371Tyr	No
40	Target NGS	.	.	.	No
41	Target NGS	.	.	.	No
42	Target NGS	.	.	.	No
43	Target NGS	.	.	.	No
44	Target NGS	PIK3CA	c.3140A>G	p.His1047Arg	No
44	Target NGS	BRCA2	13q13.1del	.	No
45	Target NGS	PIK3CA	c.3140A>G	p.His1047Arg	No
46	Target NGS	PIK3CA	c.3140A>G	p.His1047Arg	No
46	Target NGS	TP53	c.445_446delTC	p.Ser149fs	No
47	Target NGS	PTEN	c.328dup	p.Gln110fs	No
48	Target NGS	PIK3CA	c.1633G>A	p.Glu545Lys	No
49	Target NGS	.	.	.	No
50	Target NGS	PIK3CA	c.1633G>A	p.Glu545Lys	No
50	Target NGS	CHEK2	c.1111C>T	p.His371Tyr	No
51	Target NGS	.	.	.	No
52	Target NGS	PIK3CA	c.1624G>A	p.Glu542Lys	No
53	Target NGS	PTEN	c91_79+1delinesGTC	.	No
54	Target NGS	TP53	c.532_533insCCT	p.His139delinsProTyr	No
55	Target NGS	.	.	.	No
56	Target NGS	PIK3CA	c.1633G>A	p.Glu545Lys	No
57	Target NGS	PIK3CA	c.3140A>G<br>c.3140A>T	p.His1047Arg<br>p.His1047Leu	No
58	Target NGS	.	.	.	No
59	Target NGS	PIK3CA	c.1035T>A	p.Asn345Lys	No
60	Target NGS	BRCA2	c.7480C>T	p.Arg2494Ter	No

60	Target NGS	AKT1	c.49G>A	p.Glu17Lys	No
61	Target NGS	FGFR1	.	.	Yes
61	Target NGS	PTEN	10q23.31del	.	No
61	Target NGS	CCND1	.	.	Yes
62	Target NGS	.	.	.	No
63	Target NGS	FGFR1	.	.	Yes
64	Target NGS	PIK3CA	c.1633G>A	p.Glu545Lys	No
64	Target NGS	PIK3R1	c.1705_1706insTCA	p.Pro568.Asp569 insValIleLysPro	No
65	Target NGS	.	.	.	No
66	Target NGS	TP53	c.823T>C	p.Cys275Arg	No
66	Target NGS	FGFR1	.	.	Yes
66	Target NGS	PTEN	10q23.31del	.	No
66	Target NGS	FGFR2	.	.	Yes
67	Target NGS	BRCA2	c.1399A>T	p.Lys467Ter	No
67	Target NGS	PIK3CA	.	.	Yes
67	Target NGS	CCND1	.	.	Yes
68	Target NGS	PIK3CA	c.1035T>A	p.Asn345Lys	No
69	Target NGS	.	.	.	No
70	Target NGS	TP53	c.578A>G	p.His193Arg	No
71	Target NGS	.	.	.	No
72	Target NGS	PIK3CA	c.1633G>C c.3140A>G	p.Glu545Gln p.His1047Arg	No
72	Target NGS	TP53	c.1024C>T	p.Arg342Ter	No
73	Sanger sequencing	TP53	.	.	No
74	Sanger sequencing	TP53	.	.	No
75	Sanger sequencing	TP53	c.880G>T	p.E294*	No
76	Sanger sequencing	TP53	.	.	No
77	Sanger sequencing	TP53	c.880delG	p.Glu294Serfs*51	No
78	Sanger sequencing	TP53	.	.	No
79	Sanger sequencing	TP53	.	.	No
80	Sanger sequencing	TP53	.	.	No
81	Sanger sequencing	TP53	.	.	No
82	Sanger sequencing	TP53	.	.	No
83	Sanger sequencing	TP53	.	.	No
84	Sanger sequencing	TP53	.	.	No
85	Sanger sequencing	TP53	.	.	No
86	Sanger sequencing	TP53	.	.	No
87	Sanger sequencing	TP53	.	.	No
88	Sanger sequencing	TP53	c.701A>G	p.Tyr234Cys	No
89	Sanger sequencing	TP53	c.742C>T	p.Arg248Trp	No
90	Sanger sequencing	TP53	c.833C>G	p.Pro278Arg	No

91	Sanger sequencing	TP53	.	.	No
92	Sanger sequencing	TP53	.	.	No
93	Sanger sequencing	TP53	c.649delG	p.Val217Trpfs*30	No
94	Sanger sequencing	TP53	.	.	No
95	Sanger sequencing	TP53	.	.	No
96	Sanger sequencing	TP53	.	.	No
97	Sanger sequencing	TP53	.	.	No
98	Sanger sequencing	TP53	.	.	No
99	Sanger sequencing	TP53	.	.	No
100	Sanger sequencing	TP53	.	.	No
101	Sanger sequencing	TP53	.	.	No
102	Sanger sequencing	TP53	.	.	No
103	Sanger sequencing	TP53	.	.	No
104	Sanger sequencing	TP53	.	.	No
105	Sanger sequencing	TP53	.	.	No
106	Sanger sequencing	TP53	.	.	No
107	Sanger sequencing	TP53	c.782+2T>A	.	No
108	Sanger sequencing	TP53	.	.	No
109	Sanger sequencing	TP53	.	.	No
110	Sanger sequencing	TP53	.	.	No
111	Sanger sequencing	TP53	c.638G>A	p.Arg213Gln	No
112	Sanger sequencing	TP53	.	.	No
113	Sanger sequencing	TP53	.	.	No
114	Sanger sequencing	TP53	.	.	No
115	Sanger sequencing	TP53	.	.	No
116	Sanger sequencing	TP53	c.559+1G>T	.	No
117	Sanger sequencing	TP53	.	.	No
118	Sanger sequencing	TP53	.	.	No
119	Sanger sequencing	TP53	c.146delA	p.Asp49Val*fs74	No
120	Sanger sequencing	TP53	.	.	No
121	Sanger sequencing	TP53	c.430C>T	p.Gln144Ter	No
122	Sanger sequencing	TP53	.	.	No
123	Sanger sequencing	TP53	.	.	No
124	Sanger sequencing	TP53	.	.	No
125	Sanger sequencing	TP53	.	.	No
126	Sanger sequencing	TP53	.	.	No
127	Sanger sequencing	TP53	.	.	No
128	Sanger sequencing	TP53	.	.	No
129	Sanger sequencing	TP53	.	.	No
130	Sanger sequencing	TP53	.	.	No
131	Sanger sequencing	TP53	.	.	No

132	Sanger sequencing	TP53	.	.	No
133	Sanger sequencing	TP53	.	.	No
134	Sanger sequencing	TP53	.	.	No
135	Sanger sequencing	TP53	.	.	No
136	Sanger sequencing	TP53	.	.	No
137	Sanger sequencing	TP53	.	.	No
138	Sanger sequencing	TP53	.	.	No
139	Sanger sequencing	TP53	.	.	No
140	Sanger sequencing	TP53	.	.	No
141	Sanger sequencing	TP53	.	.	No

Abbreviations: cHGVS, coding DNA reference sequence Human Genome Variation Society; pHGVS, protein reference sequence Human Genome Variation Society.
