# Supplementary material for: Association between TP53 mutation and high 21-gene recurrence score in estrogen receptor-positive/HER2-negative breast cancer
Source: NPJ Breast Cancer. 2022 Feb 16;8:19. doi: 10.1038/s41523-022-00384-3 (PMC8850427; doi:10.1038/s41523-022-00384-3)
Supplement: Supplementary file 2 — Reporting Summary [file 41523_2022_384_MOESM2_ESM.pdf]

nature portfolio

Corresponding author(s): Sung Gwe Ahn

Last updated by author(s): Dec 9, 2021

Reporting Summary

Nature Portfolio wishes to improve the reproducibility of the work that we publish. This form provides structure for consistency and transparency in reporting. For further information on Nature Portfolio policies, see our Editorial Policies and the Editorial Policy Checklist.

Statistics

For all statistical analyses, confirm that the following items are present in the figure legend, table legend, main text, or Methods section.

n/a	Confirmed	
☐	☒	The exact sample size (*n*) for each experimental group/condition, given as a discrete number and unit of measurement
☐	☒	A statement on whether measurements were taken from distinct samples or whether the same sample was measured repeatedly
☐	☒	The statistical test(s) used AND whether they are one- or two-sided<br>*Only common tests should be described solely by name; describe more complex techniques in the Methods section.*
☐	☒	A description of all covariates tested
☒	☐	A description of any assumptions or corrections, such as tests of normality and adjustment for multiple comparisons
☐	☒	A full description of the statistical parameters including central tendency (e.g. means) or other basic estimates (e.g. regression coefficient) AND variation (e.g. standard deviation) or associated estimates of uncertainty (e.g. confidence intervals)
☐	☒	For null hypothesis testing, the test statistic (e.g. *F*, *t*, *r*) with confidence intervals, effect sizes, degrees of freedom and *P* value noted<br>*Give P values as exact values whenever suitable.*
☒	☐	For Bayesian analysis, information on the choice of priors and Markov chain Monte Carlo settings
☒	☐	For hierarchical and complex designs, identification of the appropriate level for tests and full reporting of outcomes
☒	☐	Estimates of effect sizes (e.g. Cohen's *d*, Pearson's *r*), indicating how they were calculated

Our web collection on statistics for biologists contains articles on many of the points above.

Software and code

Policy information about availability of computer code

Data collection	N/A
Data analysis	Statistical analyses were performed using SPSS version 25.0 (IBM Corp, Armonk, NY, USA) and R software (https://www.r-projet.org; version 3.6.1).

For manuscripts utilizing custom algorithms or software that are central to the research but not yet described in published literature, software must be made available to editors and reviewers. We strongly encourage code deposition in a community repository (e.g. GitHub). See the Nature Portfolio guidelines for submitting code & software for further information.

Data

Policy information about availability of data

All manuscripts must include a data availability statement. This statement should provide the following information, where applicable:

- Accession codes, unique identifiers, or web links for publicly available datasets
- A description of any restrictions on data availability
- For clinical datasets or third party data, please ensure that the statement adheres to our policy

Data supporting this study include clinical outcomes for which Institutional Review Board (IRB) approval is required before analysis. These data are publicly unavailable but will be made available to authorized investigators who obtain approval from the IRBs at their workplace and the Gangnam Severance Hospital, Yonsei University, Seoul, Republic of Korea. For data access requests, please contact the corresponding author, Dr. Sung Gwe Ahn. For TCGA data, we used RNA-seq data as described and normalized, log2-transformed, and median-centered expression. Information about TP53 mutations and genomic variants was downloaded from the cBioPortal. We identified PAM50-defined intrinsic subtypes as luminal A, luminal B, HER2-enriched, basal-like, and normal-like.

Field-specific reporting

Please select the one below that is the best fit for your research. If you are not sure, read the appropriate sections before making your selection.

☒ Life sciences ☐ Behavioural & social sciences ☐ Ecological, evolutionary & environmental sciences

For a reference copy of the document with all sections, see nature.com/documents/nr-reporting-summary-flat.pdf

Life sciences study design

All studies must disclose on these points even when the disclosure is negative.

Sample size	No sample size calculation was performed. This represents a retrospective study which all consecutive patients tested 21-gene recurrence score and TP53 gene sequencing were included.
Data exclusions	Those patients for whom either 21-gene recurrence score and TP53 gene sequencing results was not available were excluded.
Replication	N/A
Randomization	N/A
Blinding	N/A

Reporting for specific materials, systems and methods

We require information from authors about some types of materials, experimental systems and methods used in many studies. Here, indicate whether each material, system or method listed is relevant to your study. If you are not sure if a list item applies to your research, read the appropriate section before selecting a response.

Materials & experimental systems

n/a	Involved in the study	
☒	☐	Antibodies
☒	☐	Eukaryotic cell lines
☒	☐	Palaeontology and archaeology
☒	☐	Animals and other organisms
☐	☒	Human research participants
☐	☒	Clinical data
☒	☐	Dual use research of concern

Methods

n/a	Involved in the study	
☒	☐	ChIP-seq
☒	☐	Flow cytometry
☒	☐	MRI-based neuroimaging

Human research participants

Policy information about studies involving human research participants

Population characteristics	All relevant population characteristics were included in the manuscript.<br>All patients received primary surgery with a diagnosis of ER+/HER2- invasive breast cancer.<br>We included 141 patients with Oncotype Dx test and TP53 gene seqeuncing.<br>Median age: 48yr
Recruitment	This represents a retrospective study including all consecutive patients with 21-gene recurrence score test and TP53 gene sequencing between August 2011 and March 2020 at Gangnam Severance Hospital, Yonsei University College of Medicine, Seoul, Korea
Ethics oversight	This study was approved by the Institutional Review Board of the Gangnam Severance Hospital (3-2021-0296) and adhered to the clinical practice guidelines of the Declaration of Helsinki (2013 amendment).

Note that full information on the approval of the study protocol must also be provided in the manuscript.

Clinical data

Policy information about clinical studies

All manuscripts should comply with the ICMJE guidelines for publication of clinical research and a completed CONSORT checklist must be included with all submissions.

Clinical trial registration	N/A (This is NOT a clinical trial)
Study protocol	We retrospectively evaluated 572 patients who were surgically treated for ER+/HER2- primary invasive BC who had undergone

Study protocol	Oncotype DX® tests at Gangnam Severance Hospital, Yonsei University College of Medicine, Seoul, Korea, between August 2011 and March 2020. Among the 572 patients, we included 141 whose TP53 gene had been sequenced.
Data collection	Clinicopathological data were extracted from electronic medical records and included age, menopausal status, ER and PR status, tumor size, nodal status, HG, nuclear grade (NG), LVI, Ki-67, Oncotype DX® 21-gene RS, TP53 mutation status, and some TP53 mutation characteristics. We excluded patients diagnosed with recurrent or metachronous BC. Tumors were staged according to the 7th edition of the American Joint Committee on Cancer. Tissue sections were histologically assessed using the Elston-Ellis modification of the Scarff-Bloom-Richardson grade.
Outcomes	Distant-recurrence-free survival which is defined as the interval from curative surgery to the first distant recurrence or last censored.
